# Effect of ABCB1 SNP polymorphisms on the plasma concentrations and clinical outcomes of rivaroxaban in Chinese NVAF patients: a population pharmacokinetic-based study

**DOI:** 10.3389/fphar.2025.1574949

**Published:** 2025-04-29

**Authors:** Fei Wang, Ze Li, Youqi Huang, Qin Liu, Libin Zhao, Honghong Wang, Hongjin Gao, Mingyu Chen, Yuze Lin, Xingang Li, Min Chen

**Affiliations:** ^1^ Department of Pharmacy, Fujian Provincial Geriatric Hospital, Clinical College of Fujian Medical University, Fuzhou, China; ^2^ Department of Pharmacy, Beijing Friendship Hospital, Capital Medical University, Beijing, China; ^3^ Shengli Clinical College of Fujian Medical University, School of Pharmacy, Fujian Medical University, Fuzhou, China; ^4^ Department of Intensive Care Unit, Fujian Provincial Geriatric Hospital, Clinical College of Fujian Medical University, Fuzhou, China; ^5^ Department of Pharmacy, Liuzhou Maternity and Child Healthcare Hospital, Affiliated Maternity Hospital, Liuzhou, China; ^6^ Department of Pharmacy, Liuzhou Hospital of Guangzhou Women and Children’s Medical Center, Liuzhou, China; ^7^ Shengli Clinical College of Fujian Medical University, Department of Pharmacy, Fujian Provincial Hospital, Fuzhou University Affiliated Provincial Hospital, Fuzhou, China

**Keywords:** population pharmacokinetic, rivaroxaban, non-valvular atrial fibrillation, ABCB1 genetic polymorphisms, TDM (therapeutic drug monitoring)

## Abstract

**Background:**

This study utilized a population pharmacokinetic (PPK) approach to assess the influence of ABCB1 genetic polymorphisms on the plasma concentrations and clinical outcomes of rivaroxaban.

**Methods:**

The PPK model for rivaroxaban was developed using the nonlinear mixed-effects modelling approach and Monte Carlo simulations were employed to derive peak concentration (C_max_) and trough concentration (C_trough_). ABCB1 genetic variants were analyzed for their impact on the plasma concentrations and clinical outcomes.

**Results:**

Analysis of 287 rivaroxaban plasma concentrations from 228 non-valvular atrial fibrillation (NVAF) patients revealed significant associations between AST (aspartate aminotransferase)/ALT (alanine aminotransferase) ratios and the apparent clearance (CL/F), the apparent volume of distribution (V/F). ABCB1 1236C>T TT and ABCB1 c.2482-2236C>T CC genotypes exhibited higher dose-adjusted C_max_ (C_max_/D) compared to other relevant genotypes. Additionally, the ABCB1 3435C>T TT genotype showed lower dose-adjusted C_trough_ (C_trough_/D) compared to CC or CT genotypes. For clinical outcomes, the ABCB1 c.2482–2236C>T CC genotype had a higher bleeding risk compared to TT (RR = 1.99, 95% CI 1.08–3.69) or CT genotypes (RR = 1.42, 95% CI 1.04–1.92), and ABCB1 3435C>T TT genotype showed a higher thromboembolic risk compared to CC genotype (RR = 3.48, 95% CI 1.02–11.85).

**Conclusion:**

The PPK model incorporated CL/F and V/F with the covariate AST/ALT. Model-based simulations revealed that ABCB1 1236C>T, ABCB1 c.2482–2236C>T, and ABCB1 3435C>T genotypes had significant impacts on the plasma concentrations of rivaroxaban. Specifically, ABCB1 c.2482-2236C>T and ABCB1 3435C>T genotypes were associated with bleeding events and thromboembolic events, respectively.

## Introduction

Atrial fibrillation (AF) stands as the most prevalent sustained cardiac arrhythmia observed in clinical settings, impacting millions globally (Chugh et al., 2014). AF poses substantial risks to cardiovascular health and overall wellness. The irregular ventricular response associated with AF can lead to hemodynamic instability, significantly elevating the likelihood of thromboembolic events, particularly ischemic stroke (Chugh et al., 2014). Anticoagulant therapy, commonly using oral anticoagulants such as warfarin or non-vitamin K antagonist oral anticoagulants (NOACs), like rivaroxaban, dabigatran, or apixaban, plays a crucial role in mitigating thromboembolic risks among AF patients (January et al., 2019).

In recent years, rivaroxaban, a direct inhibitor of factor Xa, has gained considerable attention for its effectiveness and safety profile in managing thromboembolic disorders among patients with non-valvular atrial fibrillation (NVAF) (Patel et al., 2011). Despite the generally predictable nature of rivaroxaban’s PK, individual variability in drug exposure has been observed, leading to challenges in optimizing dosage regimens. This variability can impact both the efficacy and safety of rivaroxaban therapy, potentially increasing the risk of bleeding or reducing its antithrombotic effects (Testa et al., 2016). Specifically, genetic polymorphisms in drug-metabolizing enzymes and drug transporters may contribute to this variability.

Cytochrome P450 (CYP) isoforms 3A4, 3A5, and 2J2 primarily metabolize rivaroxaban (Daly and King, 2003). Investigations have indicated that the activity of CYP3A4 affects the peak concentration (C_max_) and trough concentration (C_trough_) of rivaroxaban (Sychev et al., 2018), suggesting that genetic variations in CYP genes may affect the pharmacokinetic (PK) of rivaroxaban. A recent study highlighted that individuals with mutant genotypes of CYP3A4 (rs2242480, rs2246709, and rs3735451) and CYP3A5 (rs776746) displayed higher C_trough_ of rivaroxaban compared to those with wild-type genotypes, and the minor allele (C) carriers on rs3735451 and the minor allele (A) carriers on rs2246709 were correlated with the minor bleeding events (Li X. Y. et al., 2023). Furthermore, rivaroxaban is transported by P-glycoprotein, encoded by the ATP Binding Cassette Subfamily B Member 1 (ABCB1) gene (Gnoth et al., 2011). Variations in this gene may influence P-glycoprotein activity and expression levels, thereby impacting rivaroxaban’s absorption, distribution, and elimination processes, finally leading to variations in plasma concentrations and clinical outcomes. Therefore, we aim to investigate the impact of ABCB1 gene polymorphisms on the plasma concentrations and clinical outcomes of rivaroxaban in this study.

However, accurately determining the C_max_ and C_trough_ can be challenging due to imprecise experimental operations in clinical experimental procedures. Meanwhile, Population pharmacokinetics (PPK) has emerged as a robust analytical tool for elucidating variability in drug exposure across diverse individuals within a population (Mould and Upton, 2012). PPK models can identify sources of variability and facilitate accurate prediction of PK parameters, even with limited sampling instances or variable dosing histories (Mould and Upton, 2012). In light of these considerations, the present study aims to investigate the impact of ABCB1 gene polymorphisms on the plasma concentrations and clinical outcomes of rivaroxaban in Chinese NVAF patients using a PPK-based approach. Specifically, we sought to determine whether genetic variations in ABCB1 influence the dose-adjusted C_max_ (C_max_/D) and C_trough_ (C_trough_/D) of rivaroxaban, and whether these variations are associated with bleeding or thromboembolic events. By addressing these questions, our study provides valuable insights into the role of ABCB1 genetic polymorphisms in rivaroxaban therapy, paving the way for personalized dosing strategies in NVAF patients.

## Methods

### Subjects and therapeutic interventions

This was prospective research conducted at the Fujian Provincial Hospital, which was approved by the institutional ethics committee (No. k2022-09-014). Inclusion criteria encompassed adult individuals diagnosed with NVAF receiving rivaroxaban treatment, while notable exclusion criteria included patients with poorly controlled hypertension (systolic blood pressure ≥ 160 mmHg), a history of cerebral hemorrhage or arterial dissection, or abnormal coagulation function. Specifically, poorly controlled hypertension is associated with an increased risk of bleeding events, such as intracerebral hemorrhage, which could complicate the interpretation of rivaroxaban’s safety profile (Lane and Lip, 2012). Additionally, poorly controlled hypertension may lead to secondary complications, such as renal impairment, which could indirectly influence rivaroxaban’s PK by reducing its elimination (Kvasnicka et al., 2017).

Patients received rivaroxaban doses of 5, 7.5, 10, 15, or 20 mg once daily. Blood samples were collected from residual blood after routine biochemical tests during patient clinical care, with inclusion limited to samples with validated sampling information. Plasma samples were centrifuged at 2,500×g at 4°C for 10 min and stored at −70°C. Additionally, the clinical characteristics and outcomes of the patients were extracted from medical records and the estimated glomerular filtration rate (eGFR) was calculated using the Chronic Kidney Disease Epidemiology Collaboration equation (Kong et al., 2013). Bleeding events comprised minor bleeding (epistaxis, gingival bleeding, skin ecchymosis), urinary and gastrointestinal bleeding (hematemesis, melena, stool routine positive, hematuria), and intracranial hemorrhage. Thrombotic events included arterial thrombosis (acute myocardial infarction, ischemic stroke), and venous thrombosis (lower extremity deep vein thrombosis, pulmonary embolism).

### Measurement of the collected rivaroxaban plasma concentrations

The rivaroxaban plasma concentrations were analyzed using a highly sensitive and specific high-performance liquid chromatography-tandem mass spectrometry (HPLC-MS/MS) method. This method was adapted from a previously published protocol (Derogis et al., 2017), with modifications to chromatographic conditions and the linear calibration range to suit the specific requirements of our study. Validation of the analytical method was performed in accordance with the guidelines outlined by the U.S. Food and Drug Administration (FDA) and the European Medicines Agency (EMA) for bioanalytical method validation. The calibration curve was linear over 3 ∼ 1,600 ng/mL, with an LLOQ of 3 ng/mL and r^2^ > 0.99. Both intra-day and inter-day precision and accuracy were assessed. Precision (coefficient of variation, CV) was < 5%, and accuracy ranged from 93% to 100%. Recovery was consistent and reproducible at low, medium, and high-quality control levels. No matrix interference was observed, and dilution integrity (1:2, 1:4) met the acceptance criteria. Stability studies under various conditions (short-term, freeze-thaw, long-term) were satisfactory. No carry-over was detected. Standard samples of rivaroxaban and apixaban (used as an internal standard) were obtained from Sigma-Aldrich (St. Louis, MO, United States). Methanol and formic acid of chromatography grade were sourced from Merck Company. These standards were used throughout the validation process to ensure consistency and reproducibility.

### Assessment of gene polymorphisms

Gene polymorphisms at four gene loci, specifically ABCB1 3435C>T, ABCB1 1236C>T, ABCB1 2677G>T/A, and ABCB1 c.2482-2236C>T, were investigated in this research. Massarray SNP typing technology, conducted by BGI Tech Solutions (Beijing Liuhe) Co., Limited, was employed for the detection of gene polymorphisms.

### Pharmacokinetic model development

The compartment model and nonlinear mixed-effects modelling strategy were employed in the development of the PPK model. The Phoenix^®^ NLME™ 7.0 software from Certara (St. Louis, MO) was utilized, employing the FOCE-ELS (first-order conditional estimation and extended least squares) method. It is important to note that a log-normal distribution was assumed for the inter-individual variability (IIV) of the PK parameters. Specifically, the random effect term (*η*_*i*_) associated with each individual’s parameter was assumed to follow a normal distribution with a mean of zero and a variance of *ω* ^*2*^ (Equation 1).

$$P_{i}=P\times e^{\eta_{i}}$$ (1)

where *P* is the typical value of a PK parameter and *P*_*i*_ represents the *i*th patient’s individual PK parameter.

Furthermore, the residual error is described by the proportional, additive, or combined error model (Equations 2–4).

$$C_{i}=C\times \left(1+\varepsilon_{1}\right)$$ (2)

$$C_{i}=C+\varepsilon_{2}$$ (3)

$$C_{i}=C\times \left(1+\varepsilon_{1}\right)+\varepsilon_{2}$$ (4)

where Ci and C represent the individual plasma concentrations and predictions of plasma concentrations, respectively. ε_1_ and ε_2_ represent the proportional errors and additive errors of predictions for drug concentrations, respectively, which are normally distributed with a mean of zero and a variance of σ^2^.

Interindividual variability in rivaroxaban PK was estimated, and covariate analyses were performed to identify factors that could explain or reduce this variability. The dataset included both categorical covariates and continuous covariates. The impact of categorical covariates on each parameter was assessed using a scale model (Equation 5) (Liu et al., 2022), whereas continuous covariates were evaluated using an exponential function model (Equation 6) (Li et al., 2016).

$$P_{i}=\left\{\begin{array}{c} P\times \theta_{1}\;\left(\text{Category }1\right)\\ P\times \theta_{2}\;\left(\text{Category }2\right)\\ P\times \theta_{3}\;\left(\text{Category }3\right) \end{array}\right.$$ (5)

$$P_{i}=P\times {\left(\frac{Cov}{{Cov}_{median}}\right)}^{\theta }\times e^{\eta_{i}}$$ (6)

where *Cov* and *Cov*_*median*_ represent the individual and median values of a covariate, respectively, while *θ* represents the estimated value of the continuous covariate effect. *θ*_1_, *θ*_2_ and *θ*_3_ represent the estimated values of the different categorical covariate effects, respectively.

The correlation between covariates and the parameters of the base model was used to construct the initial population model. The final population model was obtained through the forward inclusion-backward elimination approach. A covariate was deemed significant if its inclusion resulted in a reduction of more than 6.635 in the objective function value (OFV) (P < 0.01), and its exclusion led to an increase of more than 10.828 in the OFV (P < 0.001).

### Model validation

The final model’s reliability was evaluated using visual assessment methods, specifically employing goodness-of-fit (GOF) plots (Li Z. et al., 2023). These primary GOF plots included four scatter plots: conditional weighted residual errors (CWRES) versus time after last dose (TAD), CWRES versus population-predicted concentration (PRED), observations versus PRED, and observations versus individual predicted concentration (IPRED). To assess the final model’s robustness, a bootstrap resampling technique was employed. A total of 2000 datasets were generated by randomly selecting different patient combinations, and parameters were re-estimated using the final population model (Li et al., 2016). Median parameter values and their 95% confidence intervals (CI), derived from the 2.5th and 97.5th percentiles of the 2000 bootstrap-estimated parameters, were compared with the estimates of the final model. Additionally, visual predictive checks (VPCs) were performed using 1,000 simulations to assess the predictive performance of the final model (Li et al., 2015). In these VPCs, the observed drug concentrations were plotted against the 5th, 50th, and 95th percentiles of the simulated concentrations, which represent the 90% prediction intervals. Furthermore, the 90% CIs for the 5th, 50th, and 95th percentiles were calculated across the 1,000 simulated datasets to evaluate the robustness of the model predictions. Lastly, for each patient, observed plasma concentrations were plotted against time, and the corresponding predicted concentration-time profiles were generated. The individual fits were visually evaluated by assessing the alignment between observed data points and predicted concentration-time profiles, with the plots used to identify potential outliers or systematic deviations in model predictions at the individual level.

### Monte Carlo simulations

Monte Carlo simulations were performed using the final PPK model to estimate individual C_max_ and C_trough_ for each patient. A total of 1,000 simulations were conducted per patient to account for IIV and residual error. The primary objectives of the Monte Carlo simulation were: (1) to evaluate the effects of ABCB1 genetic polymorphisms on C_max_/D and C_trough_/D; and (2) to explore potential correlations between rivaroxaban plasma concentrations and clinical outcomes, including bleeding and thromboembolic events.

### Statistical analysis

The Hardy-Weinberg equilibrium test was conducted using the χ^2^ test. To evaluate the impact of ABCB1 SNPs on rivaroxaban plasma concentrations, continuous variables such as C_max_/D and C_trough_/D were compared among genotypes using the Kruskal-Wallis test. Pairwise comparisons were performed using the Mann-Whitney U test with Bonferroni correction to adjust for multiple testing. For clinical outcomes, categorical variables such as bleeding events and thromboembolic events were analyzed using the χ^2^ test or Fisher’s Exact test. Relative risks (RR) with 95% CI were calculated to assess the risk of clinical events associated with specific genotypes. All analyses were performed using Stata 17.0 (StataCorp, College Station, TX, United States). Data are presented as median and range or interquartile range, or mean ± standard deviation (SD). Statistical significance was defined as *P* < 0.05. However, for the univariate analysis, Bonferroni correction-adjusted *P*-values were considered statistically significant to address multiple testing.

## Results

### Participants

A cohort of 228 patients was successfully recruited for participation in this study, in accordance with the predetermined eligibility criteria. Throughout the study duration, a total of 287 plasma concentration data points were collected and documented. The median number of blood samples per participant was 1 (range: 1 ∼ 3; mean ± SD: 1.26 ± 0.54). Approximately 78% of participants contributed one sample, 18% contributed two samples, and 4% contributed three samples. Detailed information regarding the demographic and clinical characteristics of the enrolled patients has been summarized in Table 1. Genotyping for four SNPs was performed, and no observed frequencies deviated significantly from Hardy-Weinberg equilibrium, as shown in Table 2. Analyzing and summarizing confounding factors potentially impacting drug metabolism or clinical outcomes were conducted and presented in Table 3, where no significant differences were observed among different ABCB1 genotypes.

**TABLE 1:** Demographic and clinical characteristics of participants.

	Median (range)	Mean ± SD
Age (years)	73 (36, 94)	72.3 ± 10
Female, n (%)	88 (38.6)	
BW (kg)	65 (33.5, 99)	65.2 ± 11.2
BMI^a^ (kg/m^2^)	23.7 (13.6, 36)	24 ± 3.4
ALB (g/L)	41 (27, 54)	41 ± 4.7
BIL (μmol/L)	11.6 (2.1, 53.6)	13.6 ± 8.2
ALT (U/L)	18 (1.5, 82)	22.1 ± 13.7
AST (U/L)	21 (6.2, 197)	24.1 ± 14.2
SCR (mg/dL)	0.89 (0.31, 4.21)	0.98 ± 0.43
eGFR^b^ (mL/min)	79.2 (13.3, 130.4)	74.6 ± 20.8
CHA2DS2-VASc	4 (2, 10)	4.13 ± 1.82
HAS-BLED	2 (1, 5)	1.99 ± 0.94

BW, body weight; BMI: body mass index; ALB, albumin; BIL, bilirubin; ALT, alanine aminotransferase; AST, aspartate aminotransferase; SCR, serum creatinine; eGFR, estimated glomerular filtration rate; SD, standard deviation.

a means BMI, body weight (kg)/height (m)2.

b means eGFR, was calculated by the Chronic Kidney Disease Epidemiology Collaboration equation.

**TABLE 2:** Variation in genotypes and allele frequencies.

SNP	Genotype	n	Frequency (%)	Allele	n	Frequency (%)	*P*
ABCB1 3435C>T	CC	77	39.7	C	248	63.9	0.4822
	CT	94	48.5	T	140	36.1	
	TT	23	11.8				
ABCB1 1236C>T	CC	21	10.6	C	147	37.1	0.0557
	CT	105	53	T	249	62.9	
	TT	72	36.4				
ABCB1 2677G>T/A	GG	67	34.7	G	211	54.7	0.8048
	GT	77	39.9	T	145	37.5	
	TA	20	10.4	A	30	7.8	
	TT	24	12.4				
	AA	5	2.6				
ABCB1 c.2482–2236C>T	CC	66	33.6	C	235	59.9	0.1865
	CT	103	52.6	T	157	40.1	
	TT	27	13.8				

**TABLE 3:** Confounding factors among different ABCB1 genotypes.

SNP	Wild type	Heterozygotes mutant type	Homozygotes mutant type	*P*
ABCB1 3435C>T				
Age	72 (66, 80)	72.5 (65, 79)	74 (65, 81)	0.885
Weight	65 (60, 73)	65 (60, 72.5)	67 (55, 75)	0.968
eGFR	74.5 (56.9, 87.6)	77.6 (59.9, 91.2)	81.0 (57, 95.3)	0.424
AST/ALT	1.11 (0.91, 1.43)	1.15 (0.84, 1.46)	1.14 (0.7, 1.82)	0.953
CHA2DS2-VASc	4 (3, 5)	4 (3, 5)	4 (3, 6)	0.702
HAS-BLED	2 (1,3)	2 (1, 2)	2 (1,3)	0.860
Concomitant drug (n)				
Amiodarone	5	5	0	0.661
Hydroclopidogrel	13	12	2	0.584
Aspirin	5	6	0	0.626
Cilostazol	1	0	0	0.515
NSAIDs	0	1	0	1.000
BATCMs	9	9	1	0.661
ABCB1 1236C>T				
Age	78 (70, 84)	73 (65, 80)	72 (65, 76.5)	0.077
Weight	65 (60, 70)	65 (60, 72.5)	67 (60, 74)	0.669
eGFR	71.3 (58, 85.5)	76.4 (59.2, 88.8)	79 (57.9, 93.3)	0.753
AST/ALT	1.29 (1, 1.53)	1.19 (0.89, 1.5)	1.14 (0.79, 1.38)	0.247
CHA2DS2-VASc	5 (3, 6)	4 (3, 6)	4 (3, 5)	0.338
HAS-BLED	2 (1, 2)	2 (1, 3)	2 (1.5, 3)	0.186
Concomitant drug (n)				
Amiodarone	0	6	4	0.798
Hydroclopidogrel	3	14	12	0.877
Aspirin	1	8	3	0.821
Cilostazol	1	0	0	0.106
NSAIDs	0	1	0	1.000
BATCMs	3	9	8	0.579
ABCB1 2677G>T/A				
Age	72 (66, 79)	72 (64, 79)	74 (65, 83)	0.477
Weight	67.5 (60, 74)	64 (60, 72.5)	66 (55, 70)	0.535
eGFR	78.3 (58.7, 88.6)	78.8 (58.9, 90.1)	76.4 (59.1, 93.3)	0.590
AST/ALT	1.2 (0.89, 1.5)	1.13 (0.87, 1.45)	1.14 (0.78, 1.44)	0.899
CHA2DS2-VASc	4 (3, 5)	4 (3, 5)	4 (3, 5)	0.494
HAS-BLED	2 (1, 3)	2 (1, 2)	2 (1, 3)	0.781
Concomitant drug (n)				
Amiodarone	6	4	0	0.218
Hydroclopidogrel	14	10	5	0.246
Aspirin	3	6	2	0.409
Cilostazol	1	0	0	0.601
NSAIDs	0	1	0	1.000
BATCMs	8	9	2	0.795
ABCB1 c.2482–2236C>T				
Age	72 (67, 79)	72 (64, 79)	74 (65, 84)	0.391
Weight	65 (60, 72.5)	64 (60, 70)	69 (55, 75)	0.433
eGFR	79.3 (64.4, 88.1)	79.6 (59.2, 91.3)	68.5 (56.1, 88.6)	0.491
AST/ALT	1.23 (0.92, 1.5)	1.11 (0.87, 1.45)	1.14 (0.78, 1.67)	0.527
CHA2DS2-VASc	4 (3, 6)	4 (3, 5)	4 (3, 6)	0.711
HAS-BLED	2 (1, 3)	2 (1, 2)	2 (1, 3)	0.692
Concomitant drug (n)				
Amiodarone	6	4	0	0.150
Hydroclopidogrel	13	12	5	0.313
Aspirin	4	6	1	1.000
Cilostazol	1	0	0	0.474
NSAIDs	0	1	0	1.000
BATCMs	8	9	2	0.748

AST, aspartate aminotransferase; ALT, alanine aminotransferase; eGFR, estimated glomerular filtration rate; NSAIDs, nonsteroidal anti-inflammatory drugs; BATCMs, blood activating traditional Chinese medicines.

Values are shown as median and interquartile range.

### Population pharmacokinetic model

In this study, we evaluated six different combinations of compartment models and error models to determine the most appropriate structure for our PPK model. Specifically, we tested one-compartment and two-compartment models with additive, proportional, and combined error models. The optimal model was selected based on the OFV and diagnostic plots, with the combination yielding the lowest OFV and most consistent diagnostic plots being chosen as the final model. The OFV values for each combination are presented in Table 4. Through this comparative analysis, a one-compartment model with a proportional error model was ultimately identified as the best-fitting model for describing the PK of rivaroxaban in the studied population. Additionally, the absorption rate constant (k_a_) was fixed at 0.617 h^-1^ based on a prior PPK study involving Japanese patients (Kaneko et al., 2013). This decision was made due to the limited sampling during the absorption phase in our dataset, which could lead to high uncertainty in k_a_ estimation. Fixing k_a_ ensured stability in parameter estimation while maintaining consistency with prior knowledge.

**TABLE 4:** Objective function values for different combinations of compartment models and error models.

	Error model			
		Proportional	Additive	Combined
Compartment model	One-compartment	2,661	3,203	2,886
	Two-compartment	2,693	6,996	OOR

OOR, out of range.

A comprehensive set of covariates was evaluated for their potential influence on the apparent clearance (CL/F) and apparent volume of distribution (V/F) of rivaroxaban. The covariates analyzed included age, sex, body weight (BW), body mass index (BMI), albumin (ALB), bilirubin (BIL), alanine aminotransferase (ALT), aspartate aminotransferase (AST), the AST/ALT, ratio, serum creatinine (S_CR_), and estimated glomerular filtration rate (eGFR). Notably, the k_a_ was fixed at 0.617 h^−1^ based on findings from prior studies, thereby precluding the need for covariate analysis related to k_a_.

Covariate analysis revealed significant associations between several variables and the PK parameters of rivaroxaban. Specifically, age, AST, ALT, eGFR, and the AST/ALT ratio demonstrated notable effects on CL/F or V/F, as evidenced by diagnostic covariate plots. Consequently, these covariates were incorporated into the initial PPK model. A stepwise approach, involving forward inclusion and backward elimination, was utilized to refine the model. This iterative process identified AST/ALT ratios as the sole covariate significantly influencing CL/F and V/F of rivaroxaban. The refinement resulted in a decrease in the OFV value from 2,661 to 2,506. Consequently, the final PPK model, which incorporates the AST/ALT covariate, is represented mathematically by Equations 7, 8.

$$CL/F\left(L/h\right)=5.64\times {\left(\frac{AST/ALT}{1.188}\right)}^{-0.074}$$ (7)

$$V/F\left(L\right)=41.7\times {\left(\frac{AST/ALT}{1.188}\right)}^{0.213}$$ (8)

In Equations 7, 8, the typical values for CL/F and V/F are 5.64 L/h and 41.7 L, respectively, while the median value of AST/ALT is 1.188. The coefficients (*f*_CL/F-AST/ALT_) and (*f*_V/F-AST/ALT_) are −0.074 and 0.213, respectively, indicating the relationship between AST/ALT and these two PK parameters. The equations indicate a clear decrease in CL/F and an increase in V/F as AST/ALT ratios rise. Initially, a diagonal OMEGA matrix was used to estimate the variances of the PK parameters (CL/F and V/F). To assess potential correlations between parameters, a full OMEGA variance-covariance matrix was subsequently evaluated. However, the correlation between CL/F and V/F was found to be negligible, and the inclusion of covariances did not significantly improve the model fit. Thus, a diagonal OMEGA matrix was retained in the final model to maintain parsimony.

Table 5 displays the estimates, relative standard errors (RSE), IIV, and shrinkage of the final PPK model, alongside the residual errors. It is noteworthy that the estimates’ precision is acceptable, given the RSE values ranging from 5.49% to 15.87%. The inter-individual variability (ETA) shrinkage values for CL/F and V/F were 15.8% and 22.4%, respectively, while the residual error (EPSILON) shrinkage value for the proportional error was 18.7%. These values are below the commonly accepted threshold of 30%, indicating that the model is well-supported by the data and the parameter estimates are robust.

**TABLE 5:** Parameter estimates and bootstrap analysis of rivaroxaban population pharmacokinetic model in the NVAF population.

Parameters (Unit)	Model estimates				Bootstrap results		
	Estimate	RSE%	IIV (CV%)	shrinkage (%)	Median	RSE%	95% CI
0.617 k_a_ (h^-1^) (Freeze)							
CL/F (L/h)	5.64	5.49	34.64	15.8	5.66	4.82	5.33 ∼ 6.02
V/F (L)	41.7	7.58	19.81	22.4	41.7	7.86	38.8 ∼ 45.0
*f*_*CL*/F-AST/ALT_	−0.074	−14.74			−0.074	−15.13	−0.044 ∼ −0.092
*f*_V/F-AST/ALT_	0.213	15.87			0.218	16.25	0.135 ∼ 0.294
Residual error (proportional error)							
σ	0.71	9.61		18.7	0.71	10.17	0.57 ∼ 0.85

k_a_, absorption rate constant; CL/F, the apparent clearance; V/F, the apparent volume of distribution; RSE, relative standard error; IIV, inter-individual variability; CV, coefficient of variation; CI, confidence interval; *f*_CL/F-AST/ALT_, coefficient representing the relationship between AST/ALT and CL/F; *f*_V/F-AST/ALT_, coefficient representing the relationship between AST/ALT and V/F; NVAF, non-valvular atrial fibrillation.

### Goodness-of-fit and model evaluation

**FIGURE 1:**
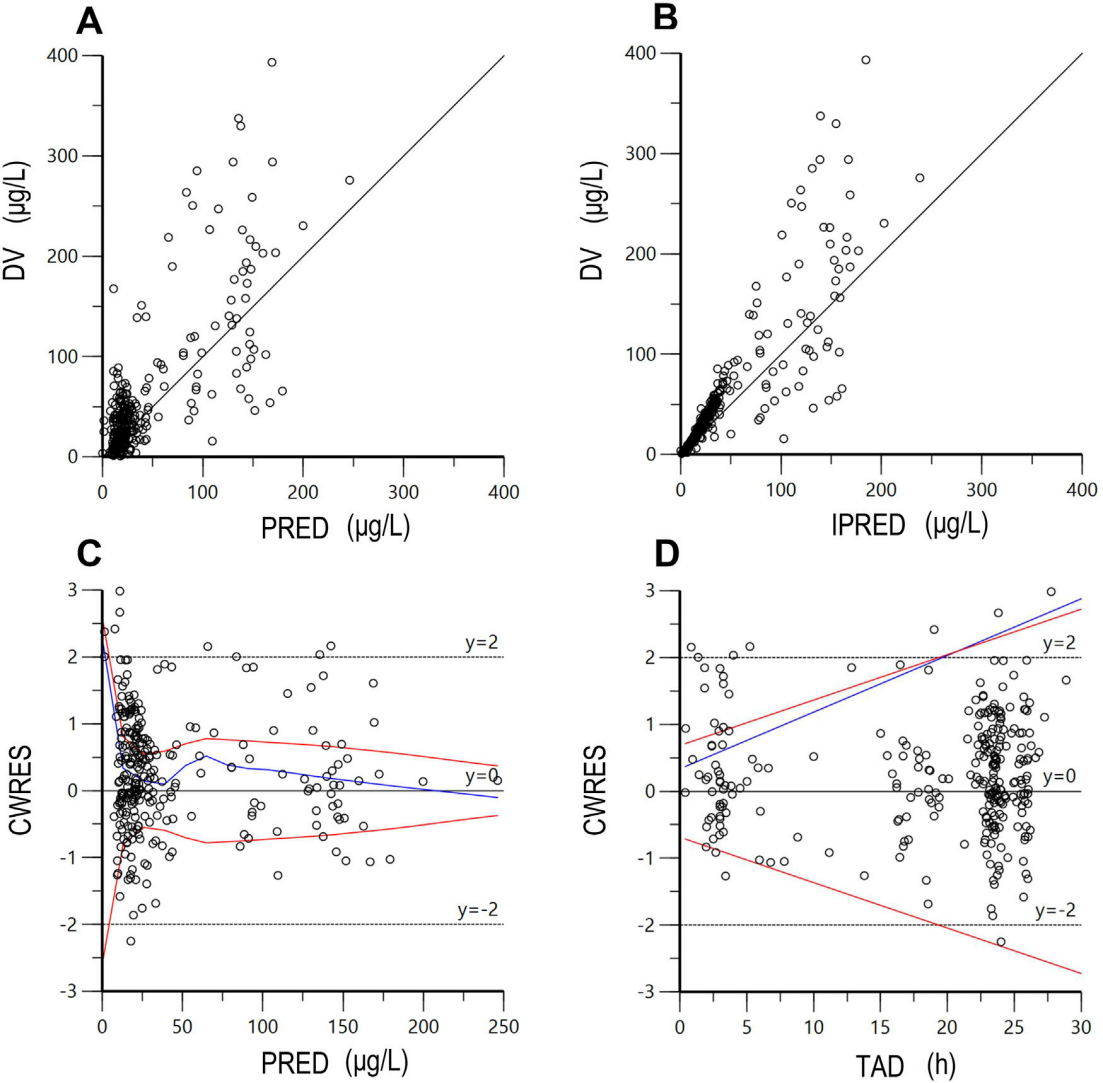
The goodness-of-fit plots of the final population pharmacokinetic model. **(A)** DV versus PRED; **(B)** DV versus IPRED; **(C)** CWRES versus PRED; **(D)** CWRES versus TAD. DV, dependent value (observations); PRED, population predicted concentration; IPRED, individual predicted concentration; CWRES, conditional weighted residuals; TAD, time after last dose. The red lines represent theoretical reference lines (y = −2, 0, +2), while the blue lines indicate the observed trends in the data.

**FIGURE 2:**
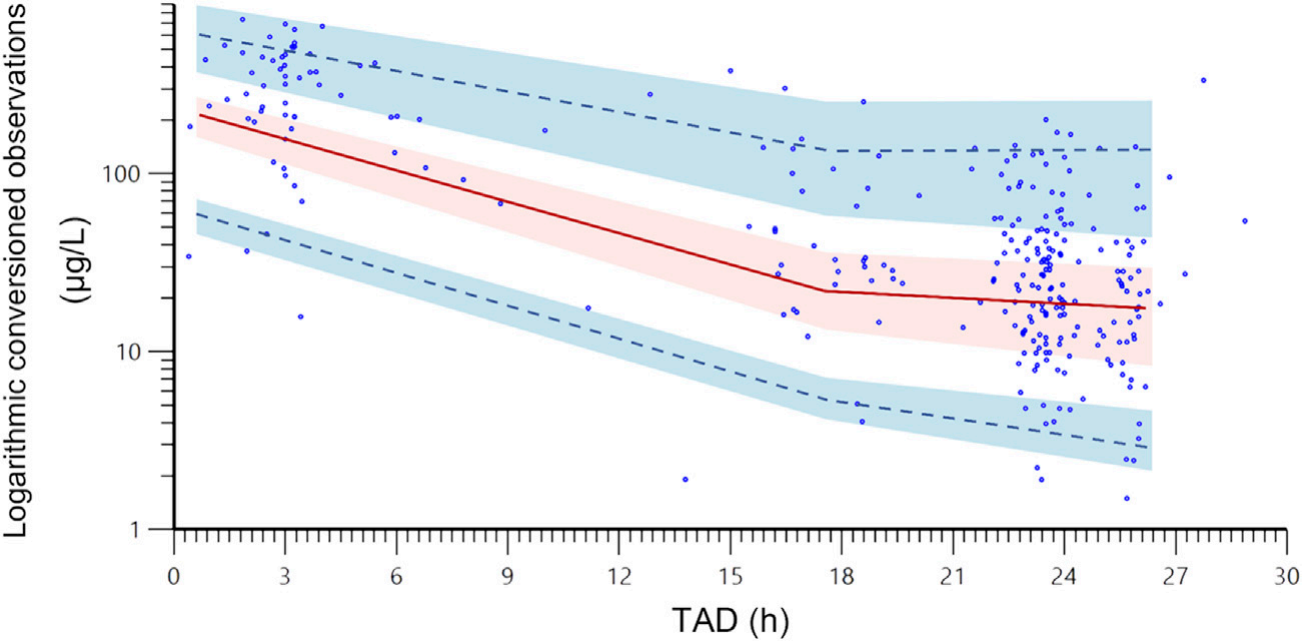
The visual predictive check plots of the final population pharmacokinetic model. The blue dots are the actual original observations; the red line is the 50% quantile from the population predicted concentrations, and the upper and lower blue dashed lines are the 95% and 5% quantiles from the population predicted concentrations, respectively; the shade areas are the 90% confidence intervals of the relevant quantiles. TAD, time after last dose.

The final PPK model underwent a favorable GOF assessment. When comparing observed concentrations (DV) with both PRED and IPRED, data points were generally distributed symmetrically around the Y = X axis, indicating good agreement between model predictions and actual observations (Figures 1A,B). However, it is worth noting that for observed concentrations above approximately 200 μg/L, the model tends to underpredict the observed values. This systematic bias may be attributed to the limited number of high-concentration data points in the dataset, which could affect the model’s ability to accurately predict extreme values. Moreover, CWRES distribution versus PRED or TAD exhibited symmetry, with the majority of values falling within the −2 to +2 range (Figures 1C,D). The reliability and robustness of the final PPK model were further confirmed through bootstrap analysis. Median parameters derived from bootstrap samples closely resembled original parameter estimates, with the 95% CI encompassing these estimates as well (Table 5). Additionally, the VPCs demonstrated that the 90% CI of predictions from the final PPK model aligned well with observed data (Figure 2). Lastly, the individual fits demonstrated strong agreement between observed and predicted concentrations across most patients, indicating that the model adequately captured the PK profiles at the individual level. Representative individual fit plots are shown in Supplementary Figure S1.

### Correlation analyses between rivaroxaban plasma concentrations and ABCB1 SNP polymorphisms

Using Monte Carlo simulations based on the final PPK model, we simulated C_max_ at 2 ∼ 4 h post-dose and C_trough_ at 24 h post-dose for each patient. Subsequently, we assessed the impact of ABCB1 SNPs on the C_max_/D and C_trough_/D of rivaroxaban. The results are summarized in Table 6, where ABCB1 1236C>T (*P* = 0.0001) and ABCB1 c.24822236C>T demonstrated (*P* = 0.0001) a significant impact on C_max_/D. Furthermore, ABCB1 3435C>T showed a significant effect on C_trough_/D (*P* = 0.0001). Further analyses in pairwise comparisons among ABCB1 SNPs revealed that ABCB1 1236C>T TT carriers exhibited higher C_max_/D values compared to CC (*P* < 0.000001) or CT (*P* < 0.000001) carriers. Similarly, ABCB1 c.2482–2236C>T CC carriers displayed higher C_max_/D values than TT (*P* = 0.000042) or CT (*P* = 0.000103) carriers. Regarding C_trough_/D, ABCB1 3435C>T TT carriers showed lower C_trough_/D compared to CC (*P* < 0.000001) or CT (*P* = 0.001744) carriers. Detailed results are provided in Table 7.

**TABLE 6:** Impacts of ABCB1 SNPs on the plasma concentrations of rivaroxaban.

SNP	Genotype	C_max_/D		*P*	C_trough_/D		*P*
ABCB1 3435C>T^a^	CC	15.80 (15.12, 16.59)		0.64424	1.36 (0.92, 1.90)		0.0001
	CT	15.86 (14.99, 16.68)			1.35 (0.94, 1.92)		
	TT	15.82 (14.45, 17.12)			1.24 (0.88, 1.73)		
ABCB1 1236C>T ^a^	CC	15.61 (14.99, 16.23)		0.0001	1.29 (0.92, 1.78)		0.23295
	CT	15.76 (14.88, 16.59)			1.33 (0.93, 1.84)		
	TT	15.97 (15.10, 16.85)			1.28 (0.90, 1.85)		
ABCB1 2677G>T/A^b^	GT	15.88 (15.06, 16.69)		0.02658	1.29 (0.90, 1.85)		0.50769
	TA	15.83 (14.67, 16.62)			1.39 (0.98 1.85)		
	GG	15.75 (15.02, 16.61)			1.30 (0.92, 1.81)		
	TT	15.96 (14.84, 17.00)			1.27 (0.85, 1.83)		
	AA	15.80 (15.22, 16.28)			1.24 (0.83, 1.82)		
ABCB1 c.2482–2236C>T^a^	CC	15.89 (15.05, 16.70)		0.0001	1.30 (0.92, 1.79)		0.4534
	CT	15.70 (15.01, 16.49)			1.30 (0.90, 1.85)		
	TT	15.69 (14.49, 16.73)			1.31 (0.88, 1.85)		

Values are shown as median and interquartile range.

^a^ represents the adjusted p-value for significance is 0.008333.

^b^ represents the adjusted p-value for significance is 0.0025.

**TABLE 7:** Pair comparisons of plasma concentrations among ABCB1 SNPs.

Plasma concentrations	SNP	Genotype pair	*P*
C_max_/D	ABCB1 1236C>T^a^	CC-CT	0.016715
		CC-TT	<0.000001
		CT-TT	<0.000001
	ABCB1 c.2482–2236C>T ^a^	CC-CT	0.000103
		CC-TT	0.000042
		CT-TT	0.123518
C_trough_/D	ABCB1 3435C>T^a^	CC-CT	0.350755
		CC-TT	<0.000001
		CT-TT	0.001744

^a^ represents the adjusted p-value for significance is 0.008333.

### Impacts of ABCB1 SNP polymorphisms on the clinical outcomes

**FIGURE 3:**
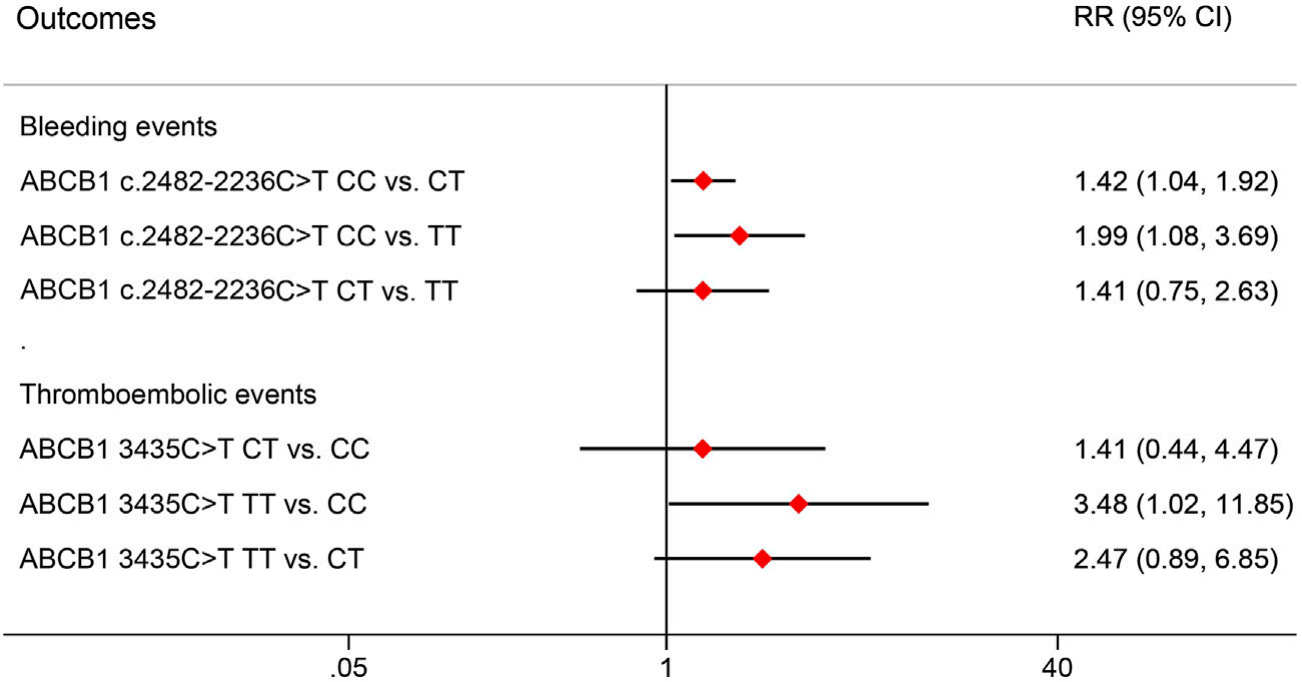
Pair comparisons of risk ratio in significant SNPs for bleeding events and thromboembolic events. RR, relative risk; CI, confidence interval; SNP, Single nucleotide polymorphism.

The effects of ABCB1 SNPs on clinical outcomes in NVAF patients receiving rivaroxaban are summarized in Table 8. We observed that ABCB1 c.2482-2236C>T and ABCB1 3435C>T were significantly associated with impacts on bleeding events and thromboembolic events, respectively. However, no difference was observed for the other ABCB1 genotypes. Further pairwise comparisons of RR were conducted for the significant SNPs and are depicted in Figure 3. The analysis revealed that ABCB1 c.24822236C>T CC carriers were at a higher risk of bleeding events compared to TT carriers (RR = 1.99, 95% CI 1.08–3.69) or CT carriers (RR = 1.42, 95% CI 1.04–1.92). Additionally, ABCB1 3435C>T TT carriers showed a higher risk of thromboembolic events compared to CC carriers (RR = 3.48, 95% CI 1.02–11.85).

**TABLE 8:** Impacts of ABCB1 SNPs on the bleeding events and thromboembolic events.

SNP	Genotype	n	Bleeding events	*P*	Thromboembolic events	*P*
ABCB1 3435C>T	CC	77	43	0.126	4	0.048
	CT	94	39		8	
	TT	23	9		5	
ABCB1 1236C>T	CC	21	13	0.105	2	0.235
	CT	105	51		12	
	TT	72	27		3	
ABCB1 2677G>T/A	GG	67	37	0.122	4	0.152
	GT	77	31		5	
	TA	20	10		3	
	TT	24	8		5	
	AA	5	4		0	
ABCB1 c.2482–2236C>T	CC	66	39	0.016	4	0.137
	CT	103	43		8	
	TT	27	8		5	

## Discussion

In this study, successful characterization of the PK of rivaroxaban was achieved using a one-compartment model. The final PPK model yielded typical values of 5.64 L/h for CL/F and 41.7 L for V/F. Through an extensive covariate search, AST/ALT ratios were identified as a significant factor affecting CL/F and V/F in the final PPK model. The relationship between CL/F, V/F and AST/ALT was best described by the exponential model, with a scaling factor (*f*_CL/F-AST/ALT_) of −0.074 and (*f*_V/F-AST/ALT_) of 0.213, indicating the decrease in CL/F and increase in V/F as AST/ALT ratios rise. Collectively, evidence from GOF analysis, bootstrap evaluation, VPCs and individual fits supports the conclusion that the final PPK model possesses the adequate predictive ability to accurately describe the PK behavior of rivaroxaban in these patients.

Prior rivaroxaban PPK studies typically excluded patients with body weight (BW) below 45 kg and severe renal or hepatic impairment (Liu et al., 2022; Li Z. et al., 2023). In contrast, our prospective study encompassed patients across a spectrum of BW (33.5 kg–99 kg), eGFR (13.3 mL/min–130.4 mL/min), and AST/ALT ratio (0.37–6.5). In our study, The AST/ALT ratio was identified as a significant covariate influencing both CL/F and V/F in the final PPK model. Beyond its statistical significance, the AST/ALT ratio has a well-established physiological relevance as an indicator of liver function (Cohen and Kaplan, 1979; Gitlin, 1982; Williams and Hoofnagle, 1988). Elevated AST/ALT ratios are associated with progressive liver impairment (Giannini et al., 1999), which can directly affect the activity of metabolic enzymes (e.g., CYP3A4, CYP3A5, and CYP2J2) and transporters (e.g., P-glycoprotein) involved in rivaroxaban disposition. Our findings suggest that higher AST/ALT ratios are associated with reduced CL/F and increased V/F, consistent with the expected effects of liver dysfunction on drug metabolism and protein binding. Incorporating the AST/ALT ratio into the PPK model thus provides valuable insights into the individualization of rivaroxaban dosing, particularly in patients with varying degrees of liver function.

Previous studies have emphasized the significant impact of genetic polymorphisms on both plasma concentration and clinical outcomes, such as bleeding or thromboembolic events, in NVAF patients undergoing rivaroxaban treatment. Xiang et al. suggested an association between SUSD3 rs76292544 and 12-month bleeding events, along with potential links of genetic variants from 52 SNPs in 36 genes (including GOT2 rs14221 and MMP13 rs640198) with the peak anti-FXa level related to bleeding events (Xiang et al., 2023). Zhang et al. found that rivaroxaban C_max_/D and ABCB1 2677G variation correlated with a higher incidence of bleeding events (Zhang et al., 2022). Additionally, Lähteenmäki et al. emphasized associations of ABCB1 3435C>T SNP and 1236T-2677T-3435T (rs1128503-rs2032582-rs1045642) haplotype with reduced thromboembolic risks in rivaroxaban users, while ABCB1 c.2482-2236C>T SNP was associated with lower bleeding risk in apixaban users (Lähteenmäki et al., 2021).

Our investigation extends these findings, highlighting the significant impacts of ABCB1 1236C>T, ABCB1 c.2482–2236C>T, and ABCB1 3435C>T on the plasma concentrations of rivaroxaban. Specifically, ABCB1 1236C>T TT and ABCB1 c.2482-2236C>T CC genotypes were associated with higher C_max_/D compared to other genotypes. Furthermore, ABCB1 3435C>T significantly influenced C_trough_/D, with ABCB1 3435C>T TT demonstrating lower C_trough_/D compared to CC or CT genotypes. Importantly, different ABCB1 genotypes showed significant differences in clinical outcomes. For instance, ABCB1 c.2482-2236C>T CC genotype had a higher bleeding risk than CT or TT genotypes, whereas ABCB1 3435C>T TT genotype showed a higher thromboembolic risk than CC genotypes.

In addition to genetic polymorphisms, various confounding factors may contribute to the variability in the plasma concentrations or clinical outcomes of rivaroxaban. Our study identified and analyzed several types of confounding factors. Firstly, rivaroxaban undergoes elimination through both renal excretion and hepatic metabolism (Kvasnicka et al., 2017), thereby age, BW, liver and kidney function were considered confounding factors. Additionally, our study identified AST/ALT ratios as a significant covariate influencing CL/F and V/F, it is important to consider the potential for confounding when interpreting the relationship between ABCB1 genotypes and rivaroxaban exposure. Specifically, differences in AST/ALT ratios across ABCB1 genotype groups could contribute to the observed genotype-exposure correlations. Secondly, CHA_2_DS_2_-VASc and HAS-BLED scores are clinical tools used to assess stroke/thromboembolism or bleeding risk in AF patients, respectively (Lane and Lip, 2012; Pisters et al., 2010). Variations in these scores among different genetic polymorphisms may lead to diverse clinical outcomes; thus, they were also included as confounding factors. In addition, polypharmacy is common among AF patients and is associated with increased mortality and bleeding risk (Proietti et al., 2016) due to drug-drug interactions (DDIs) (Ferri et al., 2022). DDIs can manifest as both pharmacokinetic and pharmacodynamic interactions. For instance, amiodarone can alter rivaroxaban plasma concentrations, while drugs like clopidogrel, aspirin, cilostazol, nonsteroidal anti-inflammatory drugs (NSAIDs), and blood-activating traditional Chinese medicines (BATCMs) can increase bleeding risk in AF patients taking rivaroxaban (Ferri et al., 2022). Therefore, these concomitant medications were considered confounding factors in our analysis. In general, the results of confounding factors analyses indicated no significant differences among different ABCB1 genotypes in the included patients (Table 3), suggesting that the observed effects of ABCB1 polymorphisms on rivaroxaban exposure are unlikely to be confounded by these factors. However, given the inherent limitations of observational studies, residual confounding cannot be entirely ruled out (Euser et al., 2009). Future studies incorporating larger sample sizes may help further disentangle the independent contributions of genetic factors.

In this study, we observed simultaneous impacts of ABCB1 genetic polymorphisms on both plasma concentrations and clinical outcomes. Specifically, carriers of the ABCB1 c.2482-2236C>T CC genotype exhibited higher C_max_/D and bleeding risk compared to those with CT or TT genotypes. On the other hand, carriers of the ABCB1 3435C>T TT genotype showed lower C_trough_/D and higher thromboembolic risk than CC carriers. These findings suggest a hypothesis that ABCB1 genotypes may influence clinical outcomes by altering the plasma concentrations of rivaroxaban. However, it is important to note that this hypothesis is based on trend observations from model simulation results. To validate this hypothesis more conclusively, a well-designed prospective study with a larger number of participants is necessary. Such a study would provide stronger evidence regarding the impact of ABCB1 genetic variants on both plasma concentrations and clinical outcomes of rivaroxaban.

### Limitation

It is important to recognize several potential limitations that could affect the applicability and precision of our findings.

Firstly, our study lacked adequate samples during the absorption phase, leading to the adoption of a fixed k_a_ value of 0.617 h^−1^ based on a previous rivaroxaban PPK study involving Japanese patients (Kaneko et al., 2013). Given potential differences between Chinese and Japanese populations (Johnson, 1997), this fixed parameter may introduce inaccuracies into the PPK model, consequently affecting the outcomes of model-based simulations.

Secondly, While the one-compartment model demonstrated superior performance in describing the PK of rivaroxaban in Chinese NVAF patients, it is important to recognize that the choice of model structure depends on the specific dataset and population characteristics. The observed biphasic decline in plasma concentrations (Figure 2) suggests that a two-compartment model might be more appropriate in certain scenarios. However, in our study, the one-compartment model provided a better fit based on OFV, while maintaining parsimony and avoiding overparameterization. Despite rigorous model development and validation procedures being followed, the potential for structural misspecification remains a limitation of the current study. Thus, the findings and conclusions of this study should be interpreted with caution.

Thirdly, our study examined individual ABCB1 SNPs’ effects on rivaroxaban plasma levels and outcomes, but it is important to note that SNPs often occur in linkage disequilibrium. Haplotypes, such as 1236T-2677T-3435T, may better explain rivaroxaban variability than single SNPs (Lähteenmäki et al., 2021). The absence of haplotype analysis is a limitation, as haplotypes capture more comprehensive genetic interactions. Future studies should explore haplotypes to clarify their combined impact on rivaroxaban exposure and risks, aiding personalized dosing strategies.

Fourthly, while we analyzed CHA_2_DS_2_-VASc and HAS-BLED scores across ABCB1 genotypes, finding no significant differences, this approach has limitations. These composite indices do not fully capture individual patient characteristics or confounders like socioeconomic status, medication adherence, or lifestyle factors, which may influence genotype-outcome associations. Our study used cross-sectional comparisons instead of time-to-event analyses (e.g., Cox models), risking bias by ignoring temporal variations in exposure or outcomes. Additionally, limited adjustments beyond composite scores may leave residual confounding unaddressed. Future studies should employ advanced methods, including time-to-event analysis and multivariable adjustments, to better assess ABCB1 polymorphisms’ effects on rivaroxaban outcomes and reduce biases.

Fifthly, Our study links ABCB1 polymorphisms to rivaroxaban plasma levels and clinical outcomes like bleeding or thromboembolic events. However, these correlations are inferred indirectly via model-based simulations, not direct pharmacokinetic-pharmacodynamic (PK-PD) relationships. A key limitation is the lack of a formal PK-PD model linking rivaroxaban exposure to outcomes. While ABCB1 polymorphisms may modulate outcomes via altered PK, results should be interpreted cautiously. Future studies with PK-PD models, integrating anti-Factor Xa activity or prothrombin time, are needed for clearer mechanistic insights.

Lastly, our study population comprised exclusively Chinese individuals, necessitating further investigations involving diverse racial backgrounds to validate the generalizability of our results. In general, given these limitations, caution should be exercised in interpreting and generalizing the conclusions drawn from our findings. Future larger and well-designed studies are warranted to corroborate and expand upon our observations.

## Conclusion

We have successfully developed a PPK model of rivaroxaban in Chinese NVAF patients, incorporating AST/ALT with CL/F and V/F. The model-based simulations indicated that ABCB1 1236C>T TT or ABCB1 c.2482-2236C>T CC genotypes exhibited higher C_max_/D compared to other genotypes, respectively. Additionally, the ABCB1 3435C>T TT genotype showed lower C_trough_/D than CC or CT genotypes. For clinical outcomes, ABCB1 c.2482-2236C>T CC genotype exhibited a higher bleeding risk than CT or TT genotypes, while ABCB1 3435C>T TT genotype demonstrated a higher thromboembolic risk than CC genotype. However, it is important to note that our conclusions should be interpreted cautiously due to inherent limitations in our study design. These findings warrant validation through larger well-designed prospective studies in the future.

## Data Availability

The original contributions presented in the study are included in the article/Supplementary Material, further inquiries can be directed to the corresponding authors.
