# Supplementary material for: Effect of ABCB1 SNP polymorphisms on the plasma concentrations and clinical outcomes of rivaroxaban in Chinese NVAF patients: a population pharmacokinetic-based study
Source: Front Pharmacol. 2025 Apr 29;16:1574949. doi: 10.3389/fphar.2025.1574949 (PMC12069994; doi:10.3389/fphar.2025.1574949)
Supplement: Supplementary file 1 [file Image1.pdf]

Title: Effect of ABCB1 SNP polymorphisms on the plasma concentrations and clinical outcomes of rivaroxaban in Chinese NVAF patients: a population pharmacokinetic-based study

A

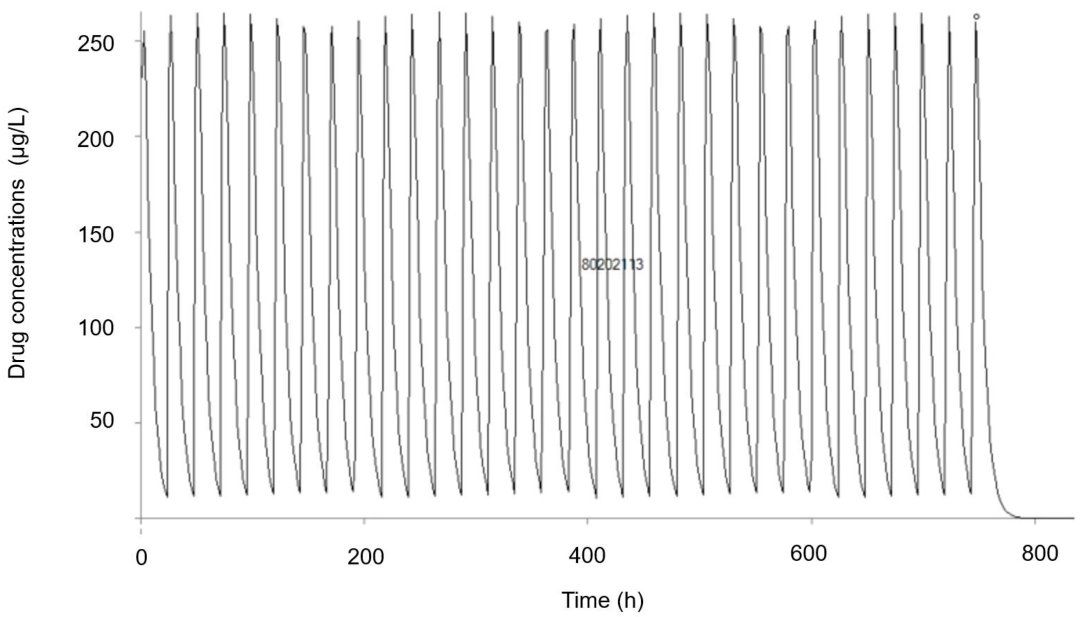

B

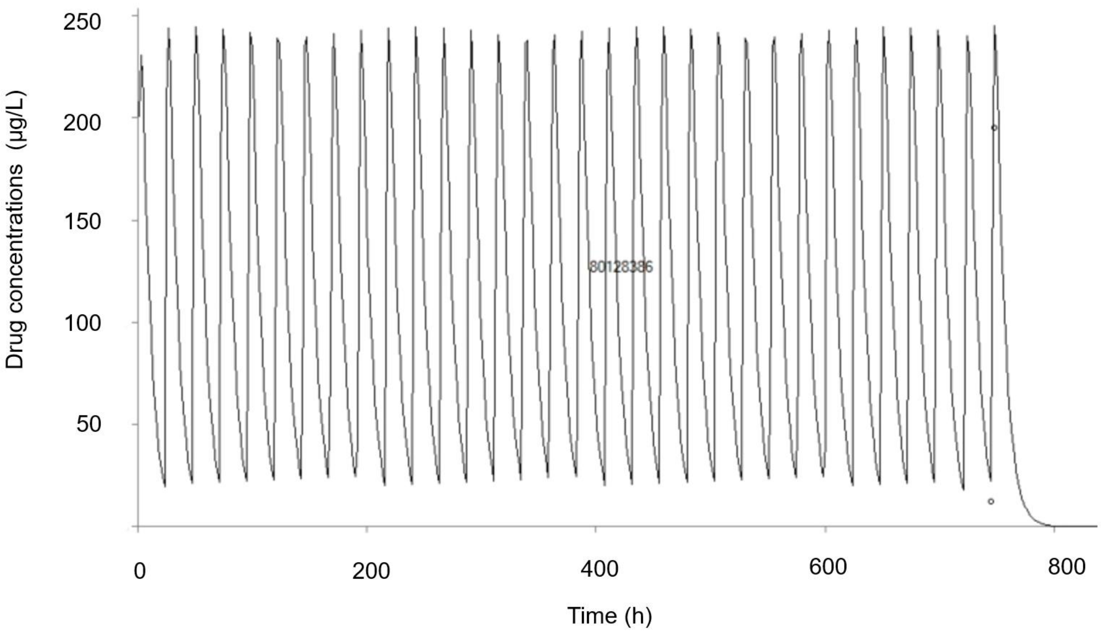

C

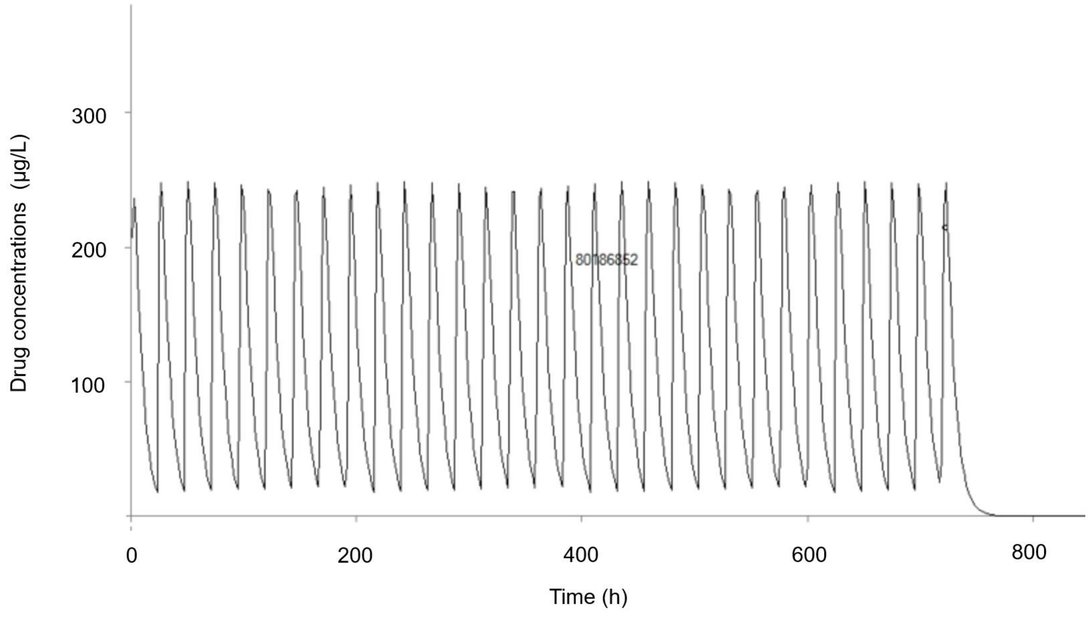

Drug concentrations (μg/L)
300
200
100
80186852
0
200
400
600
800
Time (h)

D

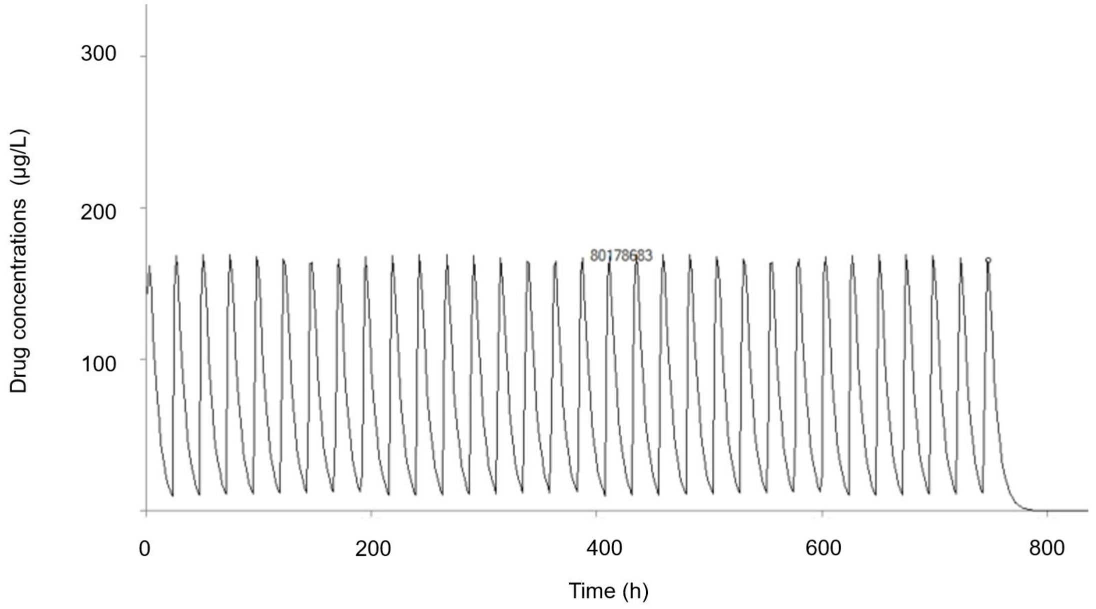

Drug concentrations (μg/L)
300
200
100
80178683
0
200
400
600
800
Time (h)

E

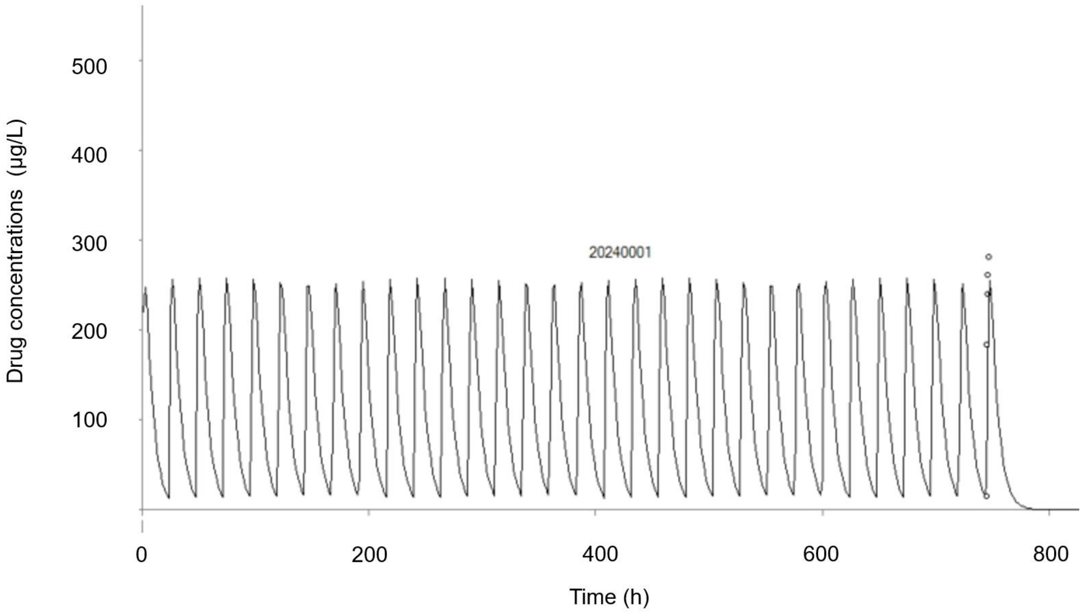

Drug concentrations (μg/L)
500
400
300
200
100
20240001
0
200
400
600
800
Time (h)

F

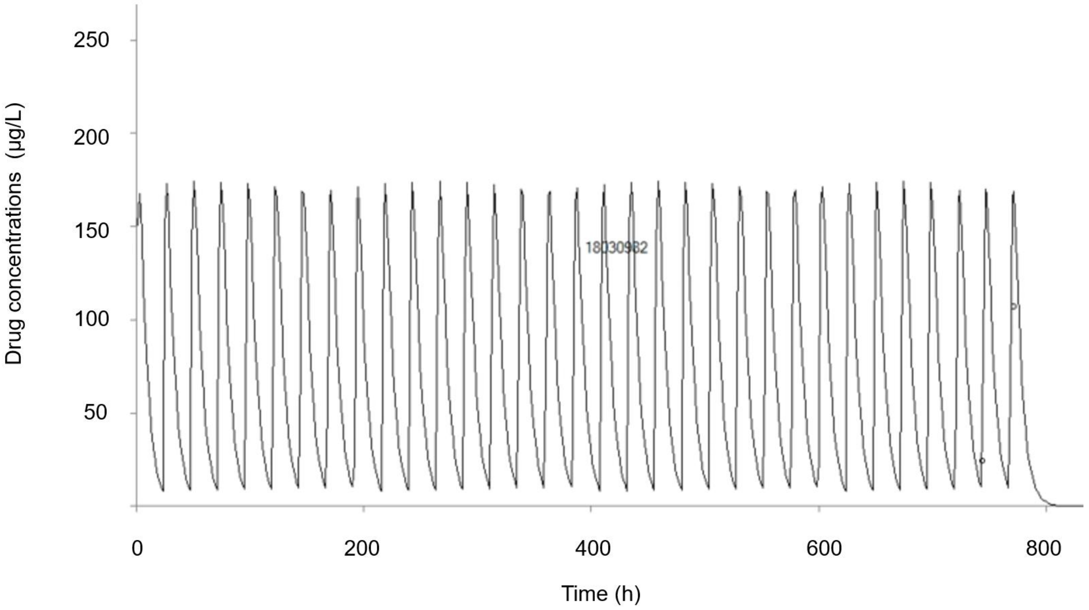

Drug concentrations (μg/L)
250
200
150
100
50
0
200
400
600
800
Time (h)

G

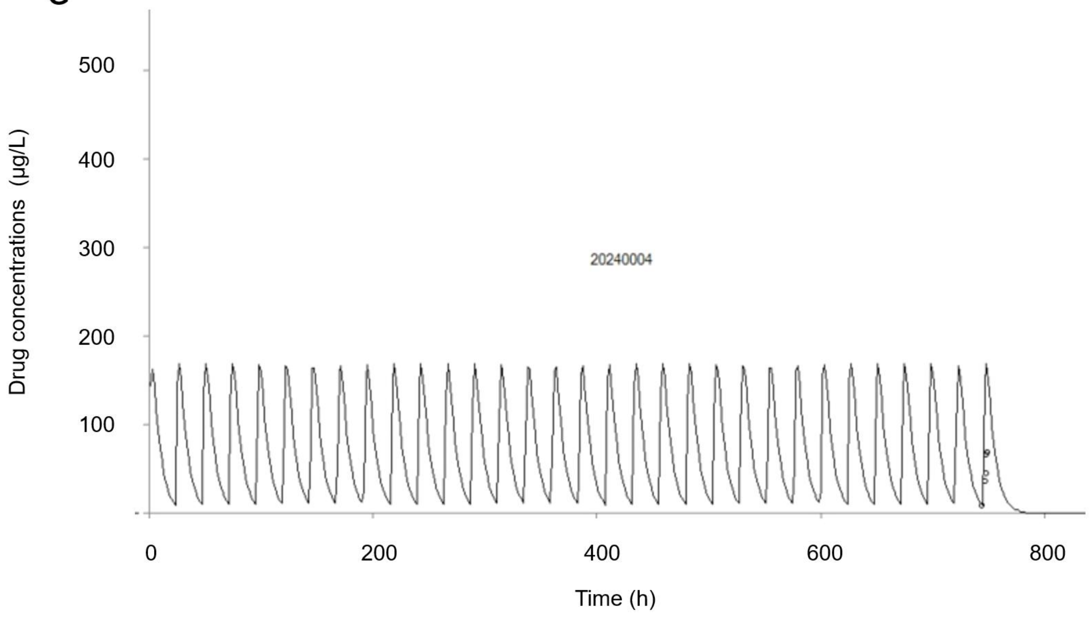

H

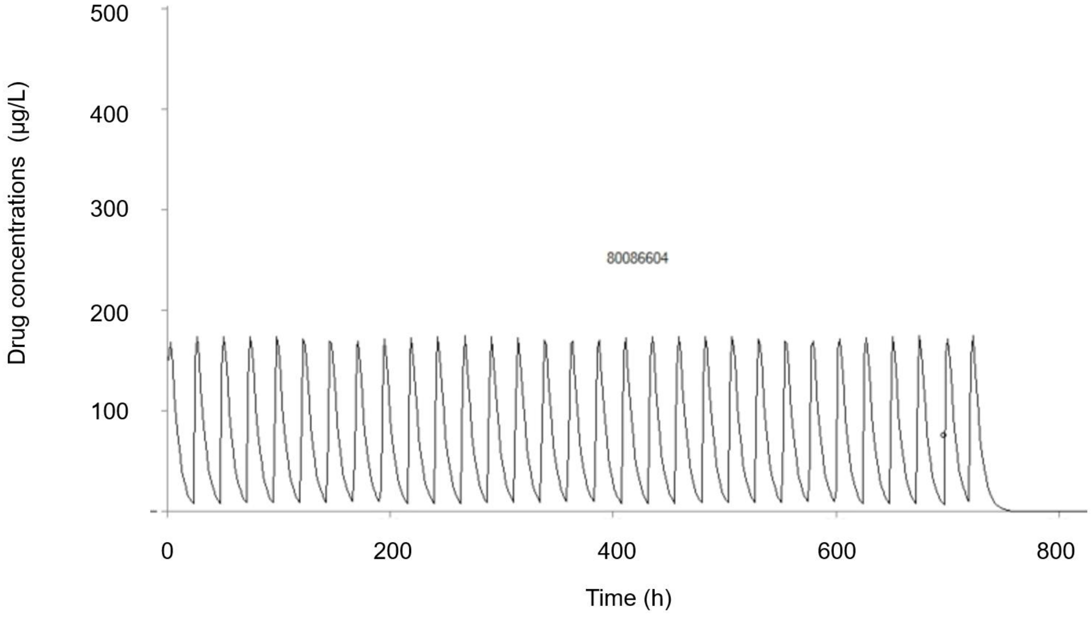

Supplementary Figure S1. Individual fits of rivaroxaban plasma concentrations for representative patients.

Black circles represent the observed rivaroxaban plasma concentrations; black curves represent the individual predicted concentration-time profiles.
